# CONNEX, a Phase III Randomized Trial Program Assessing Efficacy and Safety of Iclepertin in Schizophrenia: Recruitment and Baseline Characteristics

**DOI:** 10.1192/bjo.2024.230

**Published:** 2024-08-01

**Authors:** Zuzana Blahova, Satoru Ikezawa, Peter Falkai, John H. Krystal, Tarun Rangan

**Affiliations:** 1 Boehringer Ingelheim RCV GmbH & Co KG, Vienna, Austria; 2 Graduate School of Arts and Sciences, The University of Tokyo, Tokyo, Japan; 3 Department of Psychiatry, International University of Health and Welfare, Mita Hospital, Tokyo, Japan; 4 Clinic of Psychiatry and Psychotherapy, Ludwig Maximilians University Munich, Munich, Germany; 5 Department of Psychiatry, Yale University School of Medicine, New Haven, USA; 6 Boehringer Ingelheim, Bracknell, United Kingdom

## Abstract

**Aims:**

Glutamatergic signalling deficits contribute to the neuropathology of cognitive symptoms in schizophrenia. Iclepertin (BI 425809), a glycine transporter-1 inhibitor, enhances *N*-methyl-D-aspartate receptor signalling in the brain by increasing synaptic levels of its co-agonist glycine. The Phase III CONNEX programme aims to assess the efficacy, safety and tolerability of iclepertin in improving cognition and functioning in schizophrenia.

**Methods:**

CONNEX includes 3 randomised, double-blind, placebo-controlled parallel group trials in patients with schizophrenia from multiple centres across 41 countries in Asia, North and South America, Europe, and Asia Pacific Region (NCT04846868, NCT04846881, NCT04860830) receiving stable antipsychotic treatment. Each trial aims to recruit ~586 patients, 18–50 years old, treated with 1–2 antipsychotic medications (≥12 weeks on current drug; ≥35 days on current dose before treatment), who have functional impairment in day-to-day activities and interact ≥1 hour/week with a designated study partner. Patients with cognitive impairment due to developmental, neurological or other disorders, or receiving cognitive remediation therapy ≤12 weeks before screening will be excluded. Patients will be randomised 1:1 to once-daily oral iclepertin 10 mg (n = 293) or placebo (n = 293) for 26 weeks. Primary endpoint: change from baseline (CfB) in Measurement and Treatment Research to Improve Cognition in Schizophrenia Consensus Cognitive Battery (MCCB) overall composite T-score. Key secondary endpoints: CfB in Schizophrenia Cognition Rating Scale (SCoRS) total score and adjusted total time T-score in the Virtual Reality Functional Capacity Assessment Tool (VRFCAT).

**Results:**

Trial completion is expected in Q1 2025. By 31/01/2024, there have been 811, 699 and 655 patients screened, 533, 474 and 458 randomised, and 320, 299 and 281 who have completed the trial medication for CONNEX-1, -2 and -3, respectively. Most patients are male (CONNEX-1: 69.3%, CONNEX-2: 69.0%, CONNEX-3: 63.3%) with similar age (mean [standard deviation; SD]) (CONNEX-1: 34.0 [8.9], CONNEX-2: 35.9 [8.4], CONNEX-3: 34.0 [8.8] years). For CONNEX-1, -2 and -3, mean (SD) duration of illness is 10.6 (8.3), 12.2 (7.9) and 9.6 (7.6) years and duration of previous schizophrenia treatment is 3.9 (4.6), 4.5 (4.9) and 3.3 (4.4) years. Baseline mean (SD) MCCB overall composite T-score (1: 28.4 [13.7], 2: 27.3 [13.8], 3: 29.7 [13.7]), SCoRS total score (1: 40.5 [11.1], 2: 39.9 [9.7], 3: 38.0 [10.0]) and VRFCAT adjusted total time T-score (1: 29.6 [22.3], 2: 30.7 [20.8], 3: 33.6 [18.1]) were similar across trials.

**Conclusion:**

If successful, CONNEX will provide evidence for iclepertin as the first efficacious medication addressing cognitive impairments in schizophrenia.

Studies funded by Boehringer Ingelheim.

